# Oncological effects and complications of salvage cryotherapy for radio-recurrent prostate cancer: a systematic review and meta-analysis

**DOI:** 10.3389/fonc.2025.1534739

**Published:** 2025-04-03

**Authors:** Shengyu Zhu, Jianjiang Liu, Bin Shen, Huali Xu, Wei Zhong, Sheng Jin

**Affiliations:** ^1^ Department of Radiotherapy, Shaoxing Second Hospital, Shaoxing, Zhejiang, China; ^2^ Department of Radiotherapy, Shaoxing People’s Hospital, Shaoxing, Zhejiang, China; ^3^ Department of Cardiac Surgery, Shaoxing People’s Hospital, Shaoxing, Zhejiang, China; ^4^ Department of Urology, Shaoxing People’s Hospital, Shaoxing, Zhejiang, China; ^5^ School of Medicine, Shaoxing University, Shaoxing, Zhejiang, China

**Keywords:** prostate cancer, recurrence, radio-recurrent, salvage, cryotherapy

## Abstract

**Background:**

Cryotherapy plays a crucial role in managing radio-recurrent prostate cancer (PCa) after initial treatment. This study aims to provide a comprehensive review of its effectiveness and associated complications.

**Methods:**

A systematic review was conducted using PubMed and EMBASE databases up to June 2024, focusing on recurrence-free survival (RFS) with salvage cryotherapy across various subgroups. Severe complications were also assessed. Survival curves were reconstructed using WebPlotDigitizer and a newly developed Shiny application. The incidence of complications was summarized with a 95% confidence interval (CI) using a random-effects model. Complications were evaluated using the Clavien-Dindo Scale (CDS).

**Results:**

Thirty-six studies were included, with 15 papers (3174 patients) contributing to survival curve reconstruction. Among 1593 patients treated with salvage cryotherapy, the median RFS was 56.7 months, with 2-, 3-, and 5-year rates of 67.6%, 59.5%, and 47.3%, respectively. Factors associated with better RFS included a longer time from primary treatment to salvage therapy (TRS) [> 70 months vs. < 70 months, hazard ratio (HR) (95% CI):0.75(0.58-0.97), p=0.031], lower pre-salvage prostate-specific antigen (PSA) levels [< 5 ng/mL vs. > 5 ng/mL, HR (95% CI):0.78 (0.65-0.93), p=0.005], salvage whole-gland cryotherapy (SWC) [whole vs. focal, HR (95% CI):0.45 (0.37-0.56), p < 0.001], neoadjuvant androgen deprivation therapy (ADT) [Yes vs. No, HR (95% CI):0.79 (0.69-0.89), p < 0.001], and higher adjuvant ADT usage [16.5-34.2% vs. 0-10.5%, HR (95% CI):0.47(0.39-0.56), p < 0.001]. Concerning severe complications, 78 out of 876 patients (8.9%, 95% CI: 7-11) experienced genitourinary (GU) events, 53 out of 633 patients (8.5%, 95% CI: 6-11) suffered from urinary incontinence, 15 out of 493 patients (3.0%, 95% CI: 2-5) had urethral sloughing/stenosis, and 6 out of 522 patients (1.1%, 95% CI: 0-2) developed recto-urethral/vesical fistula. No cases of severe haematuria, urinary tract infection, or urinary retention were reported.

**Conclusions:**

Cryotherapy demonstrates a favorable safety profile and significant RFS benefits for salvage treatment of radio-recurrent PCa. Longer TRS, lower pre-salvage PSA, SWC, and peri-salvage ADT usage appear to be promising prognostic factors for RFS. However, confirmation of these findings requires randomized controlled trials (RCTs) due to the low evidence levels and study heterogeneity.

## Introduction

Approximately 30–40% of individuals diagnosed with localized prostate cancer (PCa) choose non-extirpative treatments, such as external beam radiation therapy (EBRT), brachytherapy (BT), or cryotherapy, as their primary management options (1, 2). Within this cohort, 20 to 50% are expected to experience prostate recurrence, influenced by various risk factors. A subset of these patients may benefit from salvage therapies (3–5). Salvage interventions following non-extirpative treatments include salvage radical prostatectomy (SRP), stereotactic body radiation therapy (SBRT), BT, high-intensity focused ultrasound (HIFU), and salvage cryotherapy, among others. However, due to the limited number of high-quality clinical trials and the prevalence of low-quality evidence, recommendations for their use remain inconclusive (6).

A prior meta-analysis revealed that patients undergoing salvage cryotherapy had 2-year and 5-year recurrence-free survival (RFS) rates of 68% (95% confidence interval [CI], 62–73) and 50% (95% CI, 44–56), respectively. Genitourinary (GU) complications occurred in 15% (95% CI, 10–22) of cases (7). However, the literature search for this meta-analysis was conducted up until 2019, which is relatively early. Due to space limitations, the descriptions of each study in the analysis were insufficiently detailed. Moreover, the presence of duplicate cases among the studies may have affected the accuracy of the reported results.

In our recent meta-analysis (8), we reconstructed and summarized the RFS curves and evaluated toxicities in patients with radio-recurrent PCa undergoing salvage high-dose-rate brachytherapy (HDR-BT). This analysis yielded significant subgroup findings and a summary of toxicities. However, no comparable meta-analysis has assessed salvage cryotherapy. Thus, the aim of this systematic review and meta-analysis is to evaluate the efficacy and complications of cryotherapy for radio-recurrent PCa.

## Materials and methods

### Research design

This meta-analysis adhered to the guidelines of the Preferred Reporting Items for Systematic Reviews and Meta-Analyses (PRISMA). The evaluation protocol was prospectively registered in the International Prospective Register of Systematic Reviews (PROSPERO) and is publicly available with the registration number CRD42024552270.

### Data source and searches

A comprehensive and systematic literature search was conducted across two reputable electronic databases, Embase and PubMed, covering articles from their inception through June 15, 2024. Full-text eligibility screening was independently performed by two investigators. The search strategy included the following terms: (cryotherapy OR cryosurgery OR cryoablation OR cryosurgical OR cold therapy) AND (prostate OR prostatic) AND (recurrence OR recurrent OR relapse OR salvage OR Recrudescence OR local failure OR radio-recurrent) (\**Supplementary Table 1**). Additionally, reference lists of eligible studies were manually reviewed for potential additional inclusions.

### Study selection and eligibility criteria

Inclusion criteria: 1) Patients with a confirmed diagnosis of radio-recurrent PCa; 2) Availability of quantitative data on either RFS or severe complications treated with cryotherapy, with RFS curves demonstrating rates exceeding two years.

Exclusion criteria: 1) Duplicate publications; 2) Articles lacking full-text access; 3) Non-English language publications; 4) Studies that did not employ the Clavien-Dindo Scale (CDS) to assess severe urinary complications.

Inclusion criteria for RFS curve reconstruction: 1) Fulfillment of the inclusion and exclusion criteria outlined above; 2) Availability of risk tables within the RFS curves.

Exclusion criteria for RFS curve reconstruction:1) Duplicate data.

Survival curve reconstruction was performed independently by two investigators, with any discrepancies resolved through consensus.

### Data extraction

Two investigators independently utilized a standardized data extraction form to collect relevant information, with any discrepancies addressed through discussion. Patient characteristics were categorized into two main areas: 1) Primary disease and treatment characteristics; 2) Disease and treatment details during the peri-salvage cryotherapy period. Moreover, we extracted raw data points and numbers at risk from the original studies to reconstruct individual patient data (IPD) for RFS analysis. To mitigate the impact of duplicate reports, we meticulously excluded redundant data by considering the enrollment institutions and times, ensuring the accuracy of the data related to RFS and severe complications.

### Data synthesis and analysis

The primary objective of this study is to evaluate the RFS of PCa patients treated with salvage cryotherapy across various subgroups, with a secondary focus on assessing the occurrence of severe complications.

The definition of RFS varies among studies, with the Phoenix criteria (9) being the most widely adopted standard. While some studies follow the criteria set by the American Society for Radiation Oncology (ASTRO) (10), others regard local failure, metastatic progression, or the initiation of hormone suppression therapy as indicators of recurrence. In this study, we define biochemical RFS, failure-free survival, and disease-free survival as equivalent to RFS. Urinary complications will be evaluated using the CDS, where Grade ≥ 3a is considered indicative of severe events (11).

To reconstruct survival data, screenshots of necessary survival curves and risk tables will be obtained from each publication. Raw data coordinates will then be extracted using the semi-automated tool WebPlotDigitizer. IPD will be reconstructed utilizing a novel application developed by Liu et al. (12), followed by survival curve plotting using R (version 4.0.3). Summary outcomes of complications will be presented as incidence rates with 95% CIs. These rates and CIs will be calculated using a random effects model with logit transformation, as delineated by Nyaga et al. (13), and implemented in STATA 14.0. A two-sided test will be performed, with statistical significance set at α = 0.05, and results will be considered significant if the p-value is below this threshold.

## Results

### Study selection and patient characteristics

**Figure 1:**
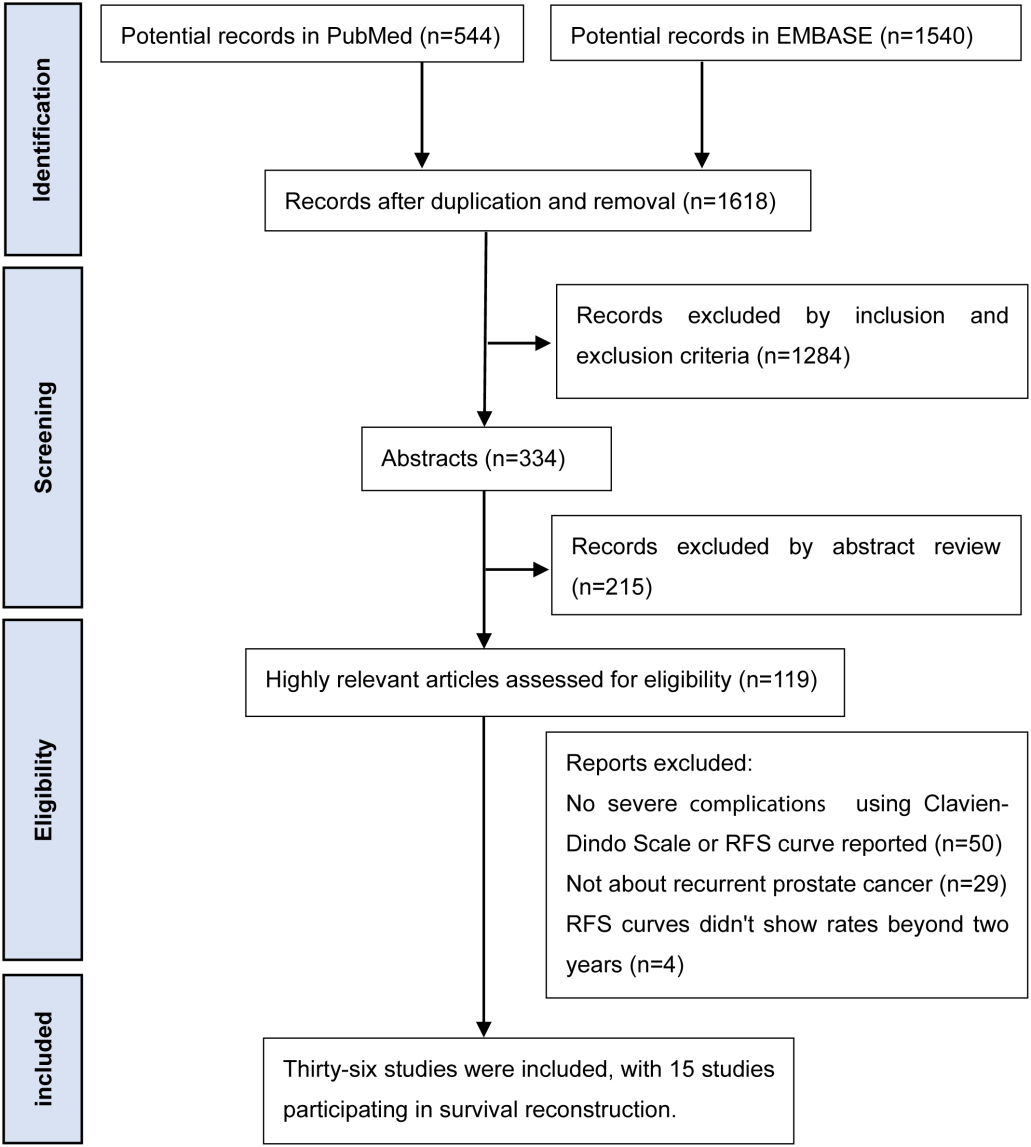
Flow chart of literature search.

After removing duplicate records, a total of 2084 entries were retrieved from two databases. A preliminary review of titles and abstracts led to the exclusion of records that did not meet the inclusion criteria, resulting in 119 records being retained (**Figure 1**). Following a comprehensive review of the full texts, 36 studies were included (14–49). Among these, curve data extraction software was used to derive 2-year or 5-year RFS rates from 34 papers (14–47) (**Supplementary Table 2**). RFS curves were reconstructed for 15 studies (16–19, 21, 26, 29–31, 37, 38, 40, 41, 43, 49), with near-complete duplication observed in 5 studies (18, 19, 26, 29, 41) (**Table 1**). A summary of severe urinary complications was compiled from 11 studies (14–17, 20, 21, 23, 27, 28, 48, 49), noting partial duplication in 2 studies (27, 49) (**Supplementary Table 3**).

**Table 1:** Details of RFS curves reconstruction for subgroups.

First author	Publication year	Patients in RFS curves (n)	Time of enrollment	Institutions of enrollment	RFS curves reconstruction or not with the influence of duplicate cases eliminated								
					Single	All	Age at recurrence	Median TRS	Pre-salvage PSA	Pre-salvage GS ≤ 7/≥8	Focal vs. Whole	Neoadjuvant ADT	Ajuvant ADT
Wimper Y (16)	2023	99	2011.5-2021.12	Radboud University Medical Center	Yes	Yes	Yes	No	Yes	Yes	Yes	No	Yes
Tan WP (17)	2023	110	2002.1-2019.9	Duke University Medical Center	Yes	Yes	Yes	Yes	Yes	Yes	Yes	No	No
Deivasigamani S (18)	2023	113	1992-2016	COLD registry; The Duke PCa database	Yes	No	No	No	No	No	No	No	No
Campbell SP (19)	2023	419	1992-2016	COLD registry; Duke Prostate Cancer databases	Yes	No	No	No	No	No	No	No	No
Exterkate L (21)	2021	169	2006-2018	Canisius-Wilhelmina Hospital	Yes	Yes	Yes	Yes	Yes	Yes	No	Yes	Yes
Bomers JGR (49)	2020	61	2011.5-2017.12	Radboud University Medical Center	Yes	No	No	Yes	No	No	No	No	No
Overduin CG (26)	2017	47	2011.5-2015.7	Radboud University Medical Center; University of Twente	Yes	No	No	No	No	No	No	No	No
Kovac E (29)	2016	486	NR	COLD registry	Yes	No	No	No	No	No	No	No	No
Li R (30) (prior ADT)	2015	254	1992.7-2014.4	COLD registry	Yes	Yes	Yes	No	Yes	Yes	Yes	Yes	Yes
Li R (30) (no prior ADT)	2015	486	1992.7-2014.4	COLD registry	Yes	Yes	Yes	No	Yes	Yes	Yes	Yes	Yes
Li YH (31) (prior ADT)	2014	26	1999-2012	COLD registry	Yes	Yes	Yes	No	Yes	Yes	Yes	Yes	No
Li YH (31) (no prior ADT)	2014	53	1999-2012	COLD registry	Yes	Yes	Yes	No	Yes	Yes	Yes	Yes	No
Philippou P (37)	2012	19	2006.2-2008.8	Barts and The London NHS Trust	Yes	Yes	Yes	Yes	Yes	Yes	Yes	No	No
Williams AK (38)	2011	176	1999-2004	University of Western Ontario	Yes	No	Yes	Yes	No	No	Yes	No	No
Spiess PE (40)	2010	277	1990.9-2005.10	Columbia University; University of Western Ontario (London); Triangle Urological Group; The University of Texas M.D. Anderson Cancer Center; Prostate Institute of America; University of California in San Francisco	Yes	Yes	No	No	Yes	Yes	No	No	Yes
Pisters LL (41)	2008	279	NR	COLD registry	Yes	No	No	No	No	No	No	No	No
lsmail M (43)	2007	100	2000.5-2005.11	The Royal Surrey County Hospital; St Luke’s Cancer Centre	Yes	Yes	Yes	No	Yes	No	Yes	No	No

RFS, Recurrence-free survival; TRS, time from primary treatment to salvage therapy; COLD, cryo online data; PSA, prostate specific antigen; GS, Gleason score; ADT, androgen deprivation therapy.

**Table 2** outlines the patient characteristics regarding primary disease and treatment across 15 studies used for the reconstruction of RFS curves. These studies, published between 2007 and 2023, included three prospective and the remainder retrospective designs. Patient enrollment occurred from 1992 to 2021, with the majority of studies conducted in North America. The number of patients enrolled in each study ranged from 19 to 486, with median pre-treatment prostate-specific antigen (PSA) levels ranging from 12 to 36 ng/mL. The distribution of Gleason score (GS) was summarized across the studies, although missing data were noted in several of them. Most patients received definitive treatment based on external beam radiation therapy (EBRT), while other treatments included BT and unspecified radiotherapy.

**Table 2:** Primary disease and treatment characteristics.

First author	Publication year	Design	Time of enrollment	Institutions of enrollment	Patients	PSA (ng/mL)	GS (%)		Primary treatment
					(n)	(range)	≤7	≥8	
Wimper Y (16)	2023	R	2011.5-2021.12	Radboud University Medical Center	99	12.5 (7.7-19.2)	NR	NR	EBRT (59.6%)/BT (24.6%)/EBRT+BT (2.6%)
Tan WP (17)	2023	R	2002.1-2019.9	Duke University Medical Center	110	NR	NR	NR	BT (29.1%)/BT+EBRT (3.6%)/EBRT (57.3%)/HDR-BT (1.8%)
Deivasigamani S (18)	2023	R	1992-2016	COLD registry; The Duke PCa database	113	NR	NR	NR	RT
Campbell SP (19)	2023	R	1992-2016	COLD registry; Duke Prostate Cancer databases	419	NR	NR	NR	RT
Exterkate L (21)	2021	R	2006-2018	Canisius-Wilhelmina Hospital	169	36 (18-66)	67.5	17.2	EBRT (37%)/EBRT+ADT (44%)/BT (18%)
Bomers JGR (49)	2020	P	2011.5-2017.12	Radboud University Medical Center	62	12.0 (7.6-18.2)	80.6	16.1	EBRT (64.5%)/BT (33.9%)/EBRT+BT (1.6%)
Overduin CG (26)	2017	R	2011.5-2015.7	Radboud University Medical Center; University of Twente	47	NR	NR	NR	EBRT (62%)/BT (36%)/EBRT+BT (2%)
Kovac E (29)	2016	R	NR	COLD registry	486	NR	NR	NR	RT
Li R (30) (prior ADT)	2015	R	1992.7-2014.4	COLD registry	254	NR	NR	NR	RT
Li R (30) (no prior ADT)	2015	R	1992.7-2014.4	COLD registry	486	NR	NR	NR	RT
Li YH (31) (prior ADT)	2014	P	1999-2012	COLD registry	32	NR	NR	NR	BT (23.1%)/ERBT (69.2%)/BT+ERBT (7.7%)
Li YH (31) (no prior ADT)	2014	P	1999-2012	COLD registry	59	NR	NR	NR	BT (41.3%)/ERBT (56.5%)/BT+ERBT (2.2%)
Philippou P (37)	2012	R	2006.2-2008.8	Barts and The London NHS Trust	19	8.67	78.9	21.1	RT (53%)/ADT+RT (47%)
Williams AK (38)	2011	R	1999-2004	University of Western Ontario	176	>10	52.4	3.7	RT
Spiess PE (40)	2010	R	1990.9-2005.10	Columbia University; University of Western Ontario (London); Triangle Urological Group; The University of Texas M.D. Anderson Cancer Center; Prostate Institute of America; University of California in San Francisco	450	17.8 (1.3-157.1)	82.7	17.3	RT
Pisters LL (41)	2008	R	NR	COLD registry	279	NR	NR	NR	BT (11.5%)/ERBT (78.1%)/beam+boost (7.2%)
lsmail M (43)	2007	P	2000.5-2005.11	The Royal Surrey County Hospital; St Luke’s Cancer Centre	100	NR	63	37	RT

R, retrospective; P, prospective; n, number; PSA, prostate specific antigen; NR, not reported; COLD, cryo online data; NR, not reported; GS, Gleason score; EBRT, external beam radiotherapy; RT, radiotherapy; HDR-BT, high-dose-rate brachytherapy; BT, brachytherapy; ADT, androgen deprivation therapy.

**Table 3** summarizes the disease and treatment characteristics during the peri-salvage cryotherapy period. In the studies reviewed, the median age at recurrence ranged from 66 to 72 years, and the median time from primary treatment to salvage therapy (TRS) ranged from < 60 to 84 months. Median pre-treatment PSA levels ranged from < 4 to 7.8 ng/mL. Imaging methods for diagnosing pelvic recurrence primarily included magnetic resonance imaging (MRI), computed tomography (CT), bone scans, and positron emission tomography-computed tomography (PET-CT). Pathological biopsies of recurrent lesions were performed for all enrolled patients, and recurrence was primarily defined according to the Phoenix criteria. The majority of studies implemented salvage whole-gland cryotherapy (SWC), though salvage focal-gland cryotherapy (SFC) was used in several cases. Additionally, the proportion of patients receiving neoadjuvant/adjuvant androgen deprivation therapy (ADT) and the follow-up time after salvage therapy were also summarized.

**Table 3:** Disease and treatment characteristics during the peri-salvage cryotherapy period.

First author	Age(years)	Median TRS (mo)	PSA (ng/mL)	GS			Imaging for relapse	Biopsy	SWC (%)	Neoadjuvant ADT (%)	Adjuvant ADT (%)	Follow-up (mo)	BCR definition
	(range)	(range)	(range)	≤7(%)	≥8(%)	≤7/≥8						(range)	
Wimper Y (16)	68 (64-72)	NR	4.2 (2.7-7.4)	44.4	38.4	1.16	MRI/PET-CT/CT	Yes	0	39.4	34.2	12	Phoenix
Tan WP (17)	67 (64.1-73.0)	78.6 (50.3-110.9)	<4	47.3	33.6	1.41	PET-CT/CT/Bone scan	Yes	100	20	NR	71(42.3-116)	Phoenix
Deivasigamani S (18)	69.1	NR	5.55 (3.8-8.9)	71.7	28.3	2.53	CT/bone scan/MRI	Yes	81.4	27.5	NR	71 (66-75)	Phoenix
Campbell SP (19)	70.9	NR	7.01	69.2	30.8	2.25	CT/Bone scan/MRI	Yes	92.1	33.9	NR	72 (60-170)	Phoenix
Exterkate L (21)	68	84(60-108)	5.5 (3.5-9.1)	53.3	30.8	1.73	MRI/PET-CT/CT	Yes	91	0	25.4	36(18-66)	Phoenix
Bomers JGR (49)	67.0 (64.0-70.8)	69.5 (49.3-95.0)	4.1 (2.5-6.8)	46.8	27.9	1.68	MRI	Yes	0	NR	NR	NR	Phoenix
Overduin CG (26)	66 (52-79)	60 (12-216)	4.9 (0.7-31.0)	51	34	1.50	MRI	Yes	0	36	NR	24(3-42)	Phoenix
Kovac E (29)	72	NR	4.7	66.5	28	2.38	NR	Yes	100	0	NR	18.2(6.4-45.2).	Phoenix
Li R (30) (prior ADT)	70 (45-88)	NR	6 (0-117.2)	55.9	36.6	1.53	NR	Yes	100	100	16.5	14.4(0-185.6)	Phoenix
Li R (30) (no prior ADT)	72 (46-93)	NR	4.7 (0-64.2)	66.5	28	2.38	NR	Yes	100	0	10.5	18.2(0.2-249.5)	Phoenix
Li YH (31) (prior ADT)	71.8	NR	7.1 (0-92.6)	78.1	21.9	3.57	NR	Yes	0	100	NR	15 (1-97)	Phoenix
Li YH (31) (no prior ADT)	70.8	NR	4.7 (0.9-19.0)	83.6	16.4	5.10	NR	Yes	0	0	NR	15 (1-97)	Phoenix
Philippou P (37)	69.2 (55-79)	72.3	6.84	78.9	21.1	3.74	MRI/bone scan	Yes	100	NR	NR	33.3	Phoenix
Williams AK (38)	>70	<60	5.0-10.0	47.1	30.5	1.54	CT/bone scan	Yes	100	NR	38.6	89.52	Phoenix
Spiess PE (40)	NR	NR	7.8 (0.5-64.2)	54.6	45.4	1.20	X-ray/CT/bone scan	Yes	NR	38.1	0	40.8(32.4-48)	Phoenix
Pisters LL (41)	70.0	NR	7.6	51.2	43.7	1.17	NR	Yes	100	NR	NR	21.6	Phoenix/ASTRO
lsmail M (43)	66.8 (54-78)	NR	5.4	NR	NR	NR	MRI/bone scan	Yes	0	46	NR	33.5(12-79)	ASTRO

NR, not reported; TRS, time from primary treatment to salvage therapy; mo, months; PSA, prostate specific antigen; GS, Gleason score; BCR, biochemical recurrence; RFS, Recurrence-free survival; MRI, magnetic resonance imaging; PET-CT, positron emission tomography; CT, computed tomography; SWC, salvage whole-gland cryotherapy; ADT, androgen deprivation therapy; ASTRO, American Society for Radiation Oncology.

### Reconstructed RFS curves for the entire cohort

**Figure 2:**
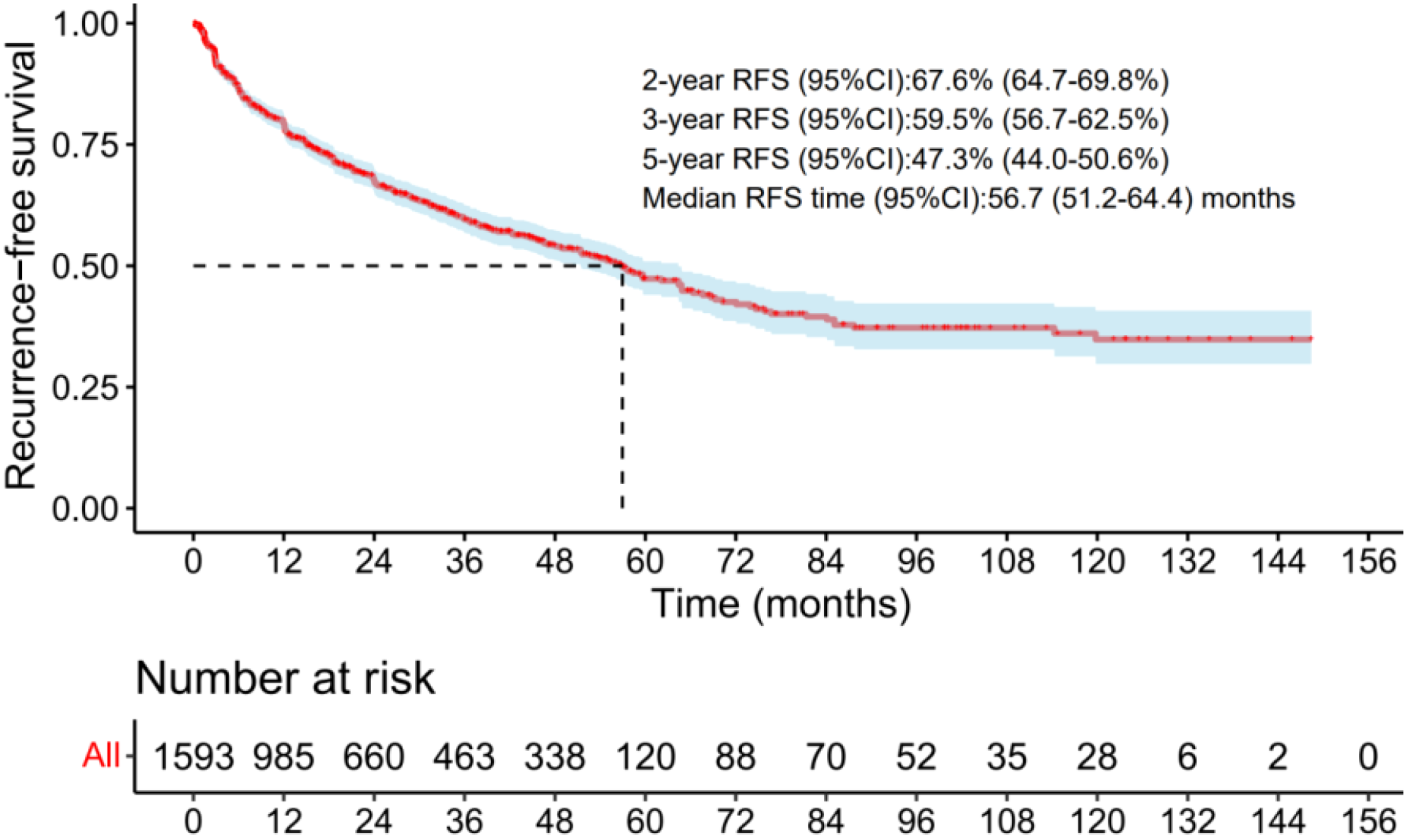
The RFS curves of the radio-recurrent patients treated with salvage cryotherapy in the total group. .

The RFS curves for the total cohort were reconstructed from data of 1,593 patients across 8 studies (16, 17, 21, 30, 31, 37, 40, 43). The median RFS duration was 56.7 months (range: 51.2–64.4 months). The pooled 2-, 3-, and 5-year RFS rates were 67.6% (95% CI: 64.7–69.8%), 59.5% (95% CI: 56.7–62.5%), and 47.3% (95% CI: 44.0–50.6%), respectively (**Figure 2**).

### Subgroup analysis of RFS

**Figure 3:**
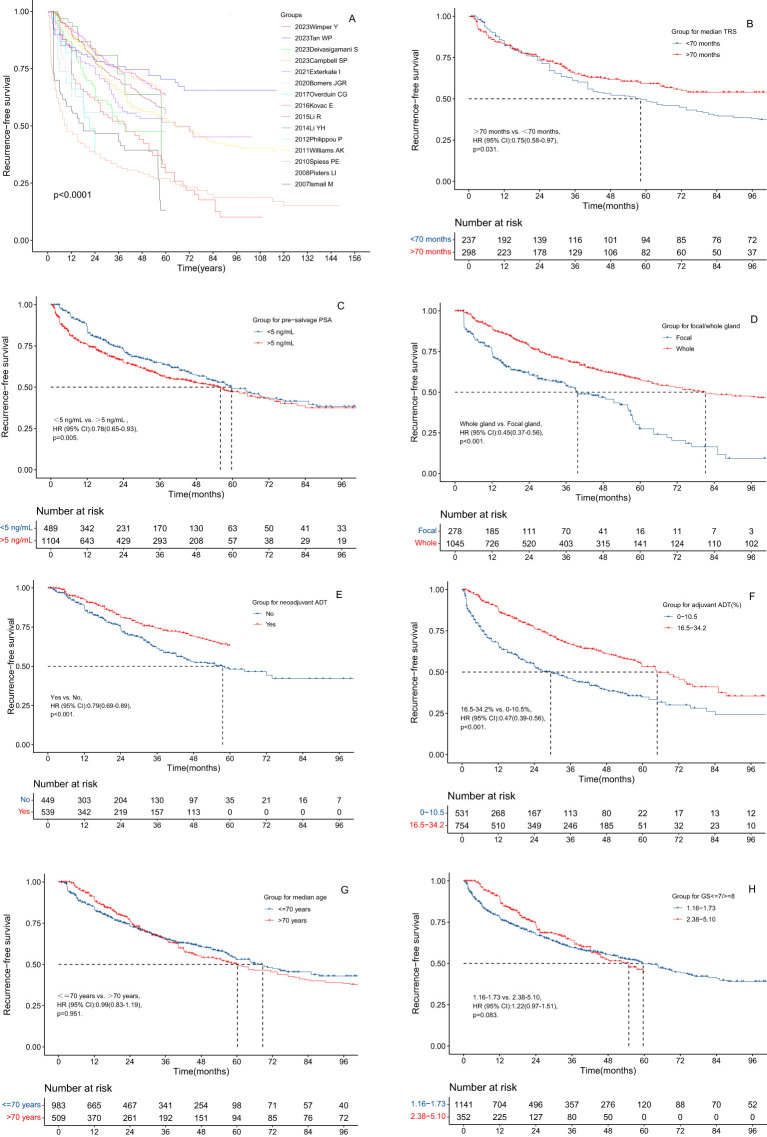
The RFS curves of the radio-recurrent patients treated with salvage cryotherapy in different subgroups. **(A)** Grouping of different papers. **(B)** Grouping of different median age at time of recurrence. **(C)** Grouping of different median TRS. **(D)** Grouping of different median pre-salvage PSA level. **(E)** Grouping of different pre-salvage values of GS ≤7/≥8. **(F)** Grouping of SFC vs. SWC. **(G)** Grouping of different median proportion of neoadjuvant ADT. **(H)** Grouping of different median proportion of adjuvant ADT.

Various factors were assessed to determine their impact on RFS following salvage cryotherapy. Patients from the studies by Campbell SP et al. (15), Tan WP et al. (17), and Kovac E et al. (29) exhibited significantly higher RFS rates compared to those in studies by Overduin CG et al. (26), Spiess PE et al. (40), and Ismail M et al. (43) (**Figure 3A**). Similarly, individuals with TRS >70 months exhibited notably higher RFS rates than those < 70 months (hazard ratio, HR: 0.75, 95% CI: 0.58-0.97, p=0.031) (**Figure 3B**). Patients with pre-salvage PSA <5 ng/mL exhibited significantly higher RFS rates compared to >5 ng/mL (HR: 0.78, 95% CI: 0.65-0.93, p = 0.005) (**Figure 3C**). Moreover, patients treated with SWC showed significantly higher RFS rates than those treated with SFC (HR: 0.45, 95% CI: 0.37-0.56, p<0.001) (**Figure 3D**). Patients who received neoadjuvant ADT exhibited significantly better RFS rates compared to those who did not (HR: 0.79, 95% CI: 0.69-0.89, p < 0.001) (**Figure 3E**). Furthermore, patients with an adjuvant ADT proportion ranging from 16.5% to 34.2% demonstrated significantly higher RFS rates than those with a proportion of 0 to 10.5% (HR: 0.47, 95% CI: 0.39-0.56, p < 0.001) (**Figure 3F**). However, no significant differences in RFS rates were observed based on median age (≤ 70 years vs. >70 years) or median Gleason score (GS ≤7 vs. GS ≥8) (**Figures 3G–H**).

### 2- and 5-year RFS rates from 34 studies

As shown in **Supplementary Table 2**, the 2-year RFS rates reported in 34 studies ranged from 15.4% to 92%, with a median rate of 72.0%. The 5-year RFS rates, available from 26 studies, ranged from 0% to 86.5%, with a median of 46.5% (13–46).

### Pooled analysis of severe complications based on the CDS

**Figure 4:**
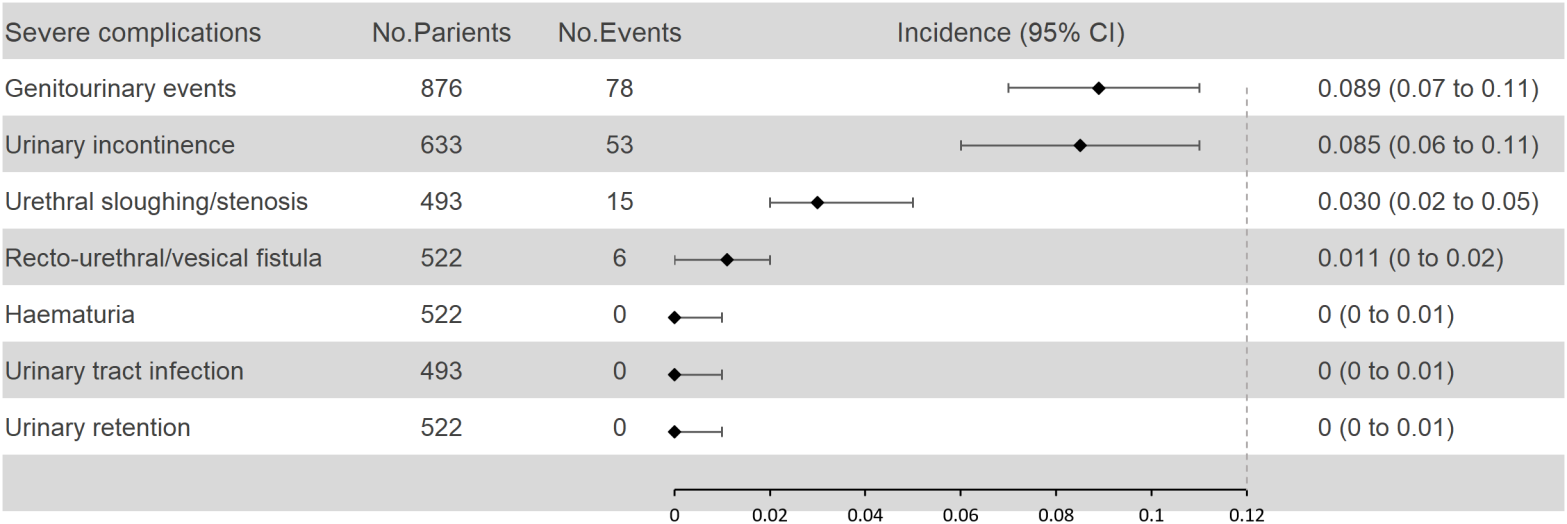
Pooled analysis of severe urinary complications according to the CDS.

**Supplementary Table 3** summarizes the severe complications reported in 11 studies (14–17, 20, 21, 23, 27, 28, 48, 49). After excluding duplicates, we compiled a summary of the most common severe complications (**Supplementary Tables 4**–**10**). Among 876 patients, 78 (8.9%, 95% CI: 7-11) experienced GU events. Of 633 patients, 53 (8.5%, 95% CI: 6-11) suffered from urinary incontinence, 15 out of 493 patients (3.0%, 95% CI: 2-5) developed urethral sloughing/stenosis, and 6 out of 522 patients (1.1%, 95% CI: 0-2) had recto-urethral or vesical fistulae (**Figure 4**). No cases of severe hematuria, urinary tract infection, or urinary retention were reported (**Figure 4**).

## Discussion

To the best of our knowledge, this meta-analysis represents the first comprehensive attempt to assess the RFS rates and the incidence of severe complications associated with salvage cryotherapy in patients with radio-recurrent PCa, utilizing a survival curve reconstruction methodology.

Our findings revealed that the estimated 2-year and 5-year RFS rates following salvage cryotherapy were 67.6% (95% CI: 64.7–69.8%) and 47.3% (95% CI: 44.0–50.6%), respectively. These rates are notably higher than those reported for salvage stereotactic body radiation therapy (SBRT), which demonstrated 2-year and 5-year RFS rates of 64.8% (95% CI: 60.3–67.9%) and 40.6% (95% CI: 34.4–46.7%) (50), but lower than those for salvage low-dose-rate brachytherapy (LDR-BT), which showed 2-year and 5-year RFS rates of 84.6% (95% CI: 81.5–87.5%) and 63.5% (95% CI: 59.0–68.7%) (51), as well as for salvage high-dose-rate brachytherapy (HDR-BT), which reported 2-year and 5-year RFS rates of 75.9% (95% CI: 72.8–79.2%) and 52.3% (95% CI: 47.5–57.4%) (8). All pooled analyses in this study utilized survival curve reconstruction techniques to ensure comparability and precision across the data.

Additionally, the aggregated incidence of severe GU complications in our cohort of 876 patients from 9 studies was 8.9% (95% CI: 7–11%). This figure was higher than the rates reported for salvage SBRT (5.8% [95% CI: 4.5–7.4%]) (50) and salvage HDR-BT (5.8% [95% CI: 4–7%]) (8), but lower than that for salvage LDR-BT (12.7% [95% CI: 10–15%]) (51). It is important to note that the CDS, employed in the current study, does not classify urinary incontinence as a graded complication. However, the incidence of severe urinary incontinence following salvage cryotherapy was non-negligible, with our pooled analysis revealing a rate of 8.5% (95% CI: 6–11%). In contrast, studies using the Common Terminology Criteria for Adverse Events (CTCAE) for toxicity assessment reported a very low incidence of severe urinary incontinence with other treatments such as SBRT, LDR-BT, and HDR-BT. Consequently, it is not possible to conclusively state that salvage cryotherapy is safer than salvage LDR-BT in terms of GU toxicity.

In the subgroup analysis of salvage cryotherapy, we identified several prognostic factors influencing RFS. Specifically, SWC and a higher proportion of ADT were associated with improved RFS rates. Given that solitary lesions are more common in localized recurrent PCa than multifocal lesions, salvage focal-gland therapy (SFC) is theoretically a viable option (52). Observational data suggest that localized ablative treatments, such as high-intensity focused ultrasound (HIFU) and cryotherapy, yield oncological outcomes comparable to those of whole-gland treatments but with reduced toxicity (53–57). In a study by Tan WP et al. (22), which included 385 patients with radio-recurrent PCa (72 of whom underwent SFC), no significant difference in 2-year progression-free survival was found between SWC and SFC (79.8% vs. 77.0%, P = 0.11) after propensity score matching. However, SFC was associated with a significantly lower rate of transient urinary retention compared to SWC (5.6% vs. 22.4%, P < 0.001) (22). Similarly, de Castro Abreu AL et al. (34) conducted a study involving 50 patients, 25 of whom were treated with SFC, and found that SWC resulted in significantly higher 5-year RFS rates compared to SFC (86% vs. 54%). Another study by Wenske S et al. (58), which included 328 patients with radio-recurrent PCa (55 of whom underwent SFC), also reported higher 5-year RFS rates for SWC compared to SFC (63% vs. 47%). Our analysis further supports these findings, showing that SWC significantly improves RFS rates relative to SFC (HR: 0.45, 95% CI: 0.37-0.56, p < 0.001). However, due to variability in baseline characteristics among the studies included in our meta-analysis, the relative effectiveness of SFC versus SWC in terms of RFS or long-term survival remains inconclusive.

In addition, the present study identified a longer duration of treatment-free survival (TRS) as a favorable prognostic factor for RFS, with patients exhibiting TRS > 70 months showing significantly better outcomes compared to those with TRS < 70 months (HR: 0.75, 95% CI: 0.58–0.97, p = 0.031). While several recent studies have investigated the effect of TRS on RFS (21, 38, 43, 59), only Exterkate L et al. (21) observed a significant association between TRS duration and RFS (HR: 0.87, 95% CI: 0.78–0.99, p = 0.03). Therefore, caution should be exercised when interpreting these results. Furthermore, our study demonstrated that the pre-salvage PSA level is a significant prognostic factor for RFS. Specifically, patients with pre-salvage PSA levels <5 ng/mL had superior RFS rates compared to those with PSA >5 ng/mL (HR: 0.78, 95% CI: 0.65–0.93, p = 0.005), although similar studies (8, 51) did not find PSA level to be predictive of RFS.

Furthermore, we found that neoadjuvant ADT prior to salvage therapy was associated with significantly higher RFS rates compared to those who did not receive neoadjuvant ADT (HR: 0.79, 95% CI: 0.69-0.89, p < 0.001). Despite extensive exploration of this issue in many studies (17, 18, 21, 26, 30, 31, 42, 48), only a few have suggested the benefits of neoadjuvant ADT for RFS (26, 30). Consequently, our findings are particularly valuable for patients with high-risk recurrent PCa, recommending peri-salvage ADT for this patient subset.

It is noteworthy that our study is the first meta-analysis to comprehensively examine the prevalence of severe GU complications, such as recto-urethral/vesical fistula, urinary incontinence, and urethral sloughing/stenosis, among others. The incidence of these severe complications was consistently low, with rates not exceeding 10%, and many instances were reported as zero. Given the findings from previous studies on salvage prostatectomy (7), our results suggest that salvage cryotherapy may offer a safer profile than salvage prostatectomy in terms of severe GU complications.

While our study provides valuable insights, it is not without its limitations. Firstly, although survival curve reconstruction for indirect comparison of survival outcomes across different treatment groups is a robust method, the homogeneity of the included studies plays a critical role in ensuring the reliability of the results. Most studies in this meta-analysis were single-arm or retrospective, with relatively low levels of evidence. Additionally, there were considerable variations in baseline patient characteristics, such as primary treatment type, pre-salvage age, median TRS, pre-salvage PSA level, pre-salvage GS, and peri-salvage ADT usage, which may have influenced the observed differences in RFS across subgroups. Furthermore, despite our efforts to avoid duplication, some studies in our analysis, particularly those from the Cryo Online Data (COLD) registry, may have overlapping patient populations. Moreover, discrepancies in data extraction tools, survival reconstruction methodologies, curve resolution techniques, and variations in researchers’ approaches could affect the accuracy of data restoration during survival reconstruction. Consequently, the reliability of our findings may be compromised, necessitating confirmation through relevant randomized controlled trials (RCTs).

## Conclusion

Cryotherapy demonstrates strong safety and offers significant benefits in RFS as salvage therapy for radio-recurrent PCa. Particularly, patients with longer TRS, lower pre-salvage PSA, SWC, and peri-salvage ADT usage experience superior RFS outcomes with minimal severe urinary complications. However, these findings require validation through RCTs due to the low evidence quality and variability across studies.
