# Supplementary material for: Oncological effects and complications of salvage cryotherapy for radio-recurrent prostate cancer: a systematic review and meta-analysis
Source: Front Oncol. 2025 Apr 3;15:1534739. doi: 10.3389/fonc.2025.1534739 (PMC12003106; doi:10.3389/fonc.2025.1534739)
Supplement: Supplementary file 1 [file Table1.docx]

**Supplementary Table 1. Search strategy and results in English from their inception to June 15, 2024.**

| Database | Search query | Results |
| --- | --- | --- |
| Pubmed | ((((((((((Cryotherapies[Title/Abstract]) OR (Cryotherapy[Title/Abstract])) OR (Cold Therapy[Title/Abstract])) OR (Cold Therapies[Title/Abstract])) OR (Cryosurgery[Title/Abstract])) OR (Cryosurgeries[Title/Abstract])) OR (Cryoablation[Title/Abstract]))) OR (cryosurgical[Title/Abstract])) AND (((prostate[Title/Abstract]) OR (PCa[Title/Abstract])) OR (Prostatic[Title/Abstract]))) AND (((((((recurrence[Title/Abstract]) OR (recurrent[Title/Abstract])) OR (Relapse[Title/Abstract])) OR (Recrudescence[Title/Abstract])) OR (salvage[Title/Abstract])) OR (local failure[Title/Abstract])) OR (radio-recurrent[Title/Abstract])) | 544 |
| Embase | 1. 'Cryotherapies' OR 'Cryotherapy' OR ' Cold Therapy' OR 'Cold Therapies' OR 'Cryosurgery' OR 'Cryosurgeries' OR 'Cryoablation' OR 'cryosurgical'  2.'Prostatic’ OR 'Prostate'  3. 'recurrence' OR 'recurrent' OR 'relapse' OR 'salvage' OR 'Recrudescence' OR 'local failure' OR 'radio-recurrent'  4. 1 and 2 and 3 | 1540 |

| Publication year and first author | PMID | Patients in RFS curves （n） | Survival reconstruction | 2-year RFS (%) | 5-year RFS (%) |
| --- | --- | --- | --- | --- | --- |
| 2024E Brito DV [14] | 38193227 | 55 | No | 81.5 | 12.1 |
| 2024Carbonell E [15] | 38478102 | 77 | No | 28.6(PSA nadir>=0.5)/84.9(PSA nadir<0.5) | 9.8(PSA nadir>=0.5)/64.3(PSA nadir<0.5) |
| 2023Wimper Y [16] | 37627122 | 99 | Yes | 61.4 | 30.1 |
| 2023Tan WP [17] | 36890104 | 110 | Yes | 78.3 | 70.9 |
| 2023Deivasigamani S [18] | 37469120 | 113 | Yes | 66.7 | 47 |
| 2023Campbell SP [19] | 37438234 | 419 | Yes | 82.3 | 63.5 |
| 2021Vestris PG [20] | 34781287 | 29 | No | 61.5 | 43,8 |
| 2021Exterkate L [21] | 33043572 | 169 | Yes | 75 | 51.7 |
| 2020Tan WP [22] | 31892490 | 385 | No | 75.5(SFC)/81.3(SWC) | NR |
| 2020Bain A [23] | 32520707 | 37 | No | 71.4 | 37.1 |
| 2019Safavy S [24] | 31050644 | 38 | No | 92(PSA nadir<=0.5)/22.4(PSA nadir>0.5) | 64.7(PSA nadir<=0.5)/0(PSA nadir>0.5) |
| 2019Barat M [25] | 31331832 | 21 | No | 65.5 | NR |
| 20170verduin CG [26] | 28409355 | 47 | Yes | 15.4(minimum iceball margin<5mm)/58.6(5-10mm)/100(>10mm) | NR |
| 2016Siddiqui KM [27] | 27157372 | 152 | No | 73.3 | 44.8 |
| 2016Lian H [28] | 27300004 | 32 | No | 78.2 | 43,.6 |
| 2016Kovac E [29] | 26915721 | 486 | Yes | 91.8(PSA nadir<0.4)/50.9(PSA nadir>=0.4) | 75.6(PSA nadir<0.4)/25.4(PSA nadir>=0.4) |
| 2015Li R [30] (prior ADT) | 25799176 | 254 | Yes | 70.8 | 44 |
| 2015Li R [30] (no prior ADT) | 25799176 | 254 | Yes | 81.6 | 63.7 |
| 2014Li YH [31] (prior ADT) | 25283814 | 26 | Yes | 89.3 | 44.8 |
| 2014Li YH [31] (no prior ADT) | 25283814 | 53 | Yes | 80.3 | 51.6 |
| 2013Spiess PE [32] | 23469778 | 132 | No | 72.6 | 45.3 |
| 2013Elkjar MC [33] | 24355453 | 39 | No | 24.2 | NR |
| 2013de Castro Abreu AL [34] | 23826840 | 50 | No | 65.5(SFC)/86(SWC) | 54.4(SFC)/86.5(SWC) |
| 2013Ahmad l [35] | 23950886 | 283 | No | 77.8(PSA nadir<=1)/27.9(PSA nadir>1) | 63.8(PSA nadir<=1) |
| 2012Spiess PE [36] | 23179729 | 156 | No | 74.1 | 46 |
| 2012Philippou P [37] | 22285952 | 19 | Yes | 55.2 | NR |
| 2011Williams AK [38] | 21185115 | 176 | Yes | 70.9 | 47.3 |
| 2011Ng CK [39] | 21251474 | 122 | No | 28.8(positive biopsy)/64(negative biopsy) | 0(positive biopsy)/30(negative biopsy) |
| 2010Spiess PE [40] | 19922545 | 277 | Yes | 37.8 | 27 |
| 2008Pisters LL [41] | 18554664 | 279 | Yes | 70.5 | 54.8 |
| 2007Ng CK [42] | 17698104 | 91 | No | 86.6(PSA<4ng/ml)/57.8(PSA=4-9.99ng/ml)/53.3(PSA>10ng/ml) | 56(PSA<4ng/ml)/29(PSA=4-9.99ng/ml)/14(PSA>10ng/ml) |
| 2007lsmail M [43] | 17662081 | 100 | Yes | 74.8 | 54.3 |
| 2005Donnelly BJ [44] | 15983627 | 46 | No | 44(biochemical faliure:PSA>=0.3)/58(biochemical faliure:PSA>=1.0) | NR |
| 2003Bahn DK [45] | 15040872 | 59 | No | 76.7(PSA threshold=0.5ng/mL)/81.5(PSA threshold=1.0ng/mL) | 59.3(PSA threshold=0.5ng/mL)/70(PSA threshold=1.0ng/mL) |
| 2001Chin JL [46] | 11371885 | 118 | No | 73.9 | NR |
| 2001Ghafar MA [47] | 11547068 | 38 | No | 73.8 | NR |
| Total | - | - | - | 72.0% (range，15.4-92%) | 46.5% (range，0-86.5%) |

**Supplementary Table 2. The 2-year and 5-year RFS rates of different papers using curve data reading software.**

RFS, Recurrence-free survival; NR, not reported; SFC, salvage focal-gland cryotherapy; SWC, salvage whole-gland cryotherapy; PSA, prostate specific antigen.

**Supplementary Table 3. Summary of severe complications according to the** **Clavien-Dindo Scale (CDS).**

| First author | Time of enrollment | Institutions of enrollment | Year | Patients  (n) | Severe GU events  (n) | Haematuria | Recto-urethral/vesical fistula | Urinary incontinence | UTI | Urinary retention | Urethral sloughing/stenosis | Duplicate reporting |
| --- | --- | --- | --- | --- | --- | --- | --- | --- | --- | --- | --- | --- |
| E Brito DV [14] | 2014.1-2022.12 | Portuguese Institute of Oncology Coimbra | 2024 | 55 | 3 | 0 | 0 | 3 | 0 | 0 | 0 | No |
| Carbonell E [15] | 2008-2020 | (Spain) Hospital Clínic de Barcelona/Parc Taulí Hospital/Hospital de Terrassa | 2024 | 77 | 4 | 0 | 0 | 3 | 0 | 0 | 0 | No |
| Wimper Y [16] | 2011.5-2021.12 | (Netherland) Radboud Institute for Health Sciences/Ziekenhuisgroep Twente | 2023 | 99 | 3 | NR | NR | NR | NR | NR | NR | No |
| Tan WP [17] | 2002.1-2019.9 | (USA)Duke University Medical Center/New York University Langone Health/Lee Mofﬁtt Cancer Center | 2023 | 110 | 3 | 0 | 1 | 0 | 0 | 0 | 0 | No |
| Vestris PG [20] | 2011.11-2019.4 | (France)Louis Hospital | 2021 | 29 | 2 | 0 | 0 | 2 | NR | 0 | NR | No |
| Exterkate L [21] | 2006-2018 | (Netherland) Canisius-Wilhelmina Hospital/niversity Medical Center Utrecht | 2021 | 169 | 2 | NR | NR | NR | NR | NR | NR | No |
| Chin JL [48] | 1992-2004 | (Canada/London/USA) University of Western Ontario/London Health Sciences Centre/The University of Texas MD Anderson Cancer Center | 2021 | 268 | 55 | NR | NR | 44 | NR | NR | NR | No |
| Bain A [23] | 2007–2017 | (USA)University of Alberta | 2020 | 37 | 4 | NR | NR | NR | NR | NR | NR | No |
| Bomers JGR [49] | 2011.5-2017.12 | (Netherland/Germany) Radboud University Medical Center/Ziekenhuisgroep Twente/Strahlentherapie Bonn Rhein Sieg (E.N.J.T.v.L.) / University of Twente | 2020 | 62 | 4 | 0 | 1 | 0 | 0 | 1 | 1 | Partially yes |
| Siddiqui KM [27] | 1995-2004 | (Canda/Saudi Arabia) Western University/University of Dammam/Woodstock Hospital | 2016 | 157 | 22 | 0 | 4 | 5 | 0 | 0 | 13 | Partially yes |
| Lian H [28] | 2006.1-2010.7 | (China) Afffliated Drum Tower Hospital of Nanjing University | 2016 | 32 | 2 | 0 | 0 | 1 | 0 | 0 | 1 | No |

n, number; GU, genitourinary; UTI, Urinary tract infection; NR, not reported.

**Supplementary Table 4. Severe genitourinary (GU) complications.**

| First author | Year | Patients(n) | Severe GU (n) | Incidence (95% CI) | LL | UL |
| --- | --- | --- | --- | --- | --- | --- |
| E Brito DV [14] | 2024 | 55 | 3 | 0.055 | 0.01 | 0.15 |
| Carbonell E [15] | 2024 | 77 | 4 | 0.052 | 0.01 | 0.13 |
| Wimper Y [16] | 2023 | 99 | 3 | 0.030 | 0.01 | 0.10 |
| Tan WP [17] | 2023 | 110 | 3 | 0.027 | 0.01 | 0.08 |
| Vestris PG [20] | 2021 | 29 | 2 | 0.069 | 0.01 | 0.23 |
| Chin JL [48] | 2021 | 268 | 55 | 0.205 | 0.16 | 0.26 |
| Exterkate L [21] | 2020 | 169 | 2 | 0.012 | 0.00 | 0.04 |
| Bain A [23] | 2020 | 37 | 4 | 0.108 | 0.03 | 0.25 |
| Lian H [28] | 2016 | 32 | 2 | 0.063 | 0.01 | 0.21 |
| Total | - | 876 | 78 | 0.089 | 0.07 | 0.11 |

CI, conﬁdence interval; LL, lower limit; UL, upper limit;

**Supplementary Table 5. Severe haematuria complications.**

| First author | Year | Patients(n) | Haematuria | Incidence (95% CI) | LL | UL |
| --- | --- | --- | --- | --- | --- | --- |
| Siddiqui KM [27] | 2016 | 157 | 0 | 0.000 | 0.00 | 0.02 |
| Lian H [28] | 2016 | 32 | 0 | 0.000 | 0.00 | 0.11 |
| Vestris PG [20] | 2021 | 29 | 0 | 0.000 | 0.00 | 0.12 |
| Bomers JGR [49] | 2020 | 62 | 0 | 0.000 | 0.00 | 0.06 |
| Carbonell E [15] | 2024 | 77 | 0 | 0.000 | 0.00 | 0.05 |
| Tan WP [17] | 2023 | 110 | 0 | 0.000 | 0.00 | 0.03 |
| E Brito DV [14] | 2024 | 55 | 0 | 0.000 | 0.00 | 0.06 |
| Total | - | 522 | 0 | 0.000 | 0.00 | 0.01 |

CI, conﬁdence interval; LL, lower limit; UL, upper limit;

**Supplementary Table 6. Severe recto-urethral/vesical fistula complications.**

| First author | Year | Patients(n) | Recto-urethral/vesical fistula | Incidence (95% CI) | LL | UL |
| --- | --- | --- | --- | --- | --- | --- |
| Siddiqui KM [27] | 2016 | 157 | 4 | 0.025 | 0.01 | 0.06 |
| Lian H [28] | 2016 | 32 | 0 | 0.000 | 0.00 | 0.11 |
| Vestris PG [20] | 2021 | 29 | 0 | 0.000 | 0.00 | 0.12 |
| Bomers JGR [49] | 2020 | 62 | 1 | 0.016 | 0.00 | 0.09 |
| Carbonell E [15] | 2024 | 77 | 0 | 0.000 | 0.00 | 0.05 |
| Tan WP [17] | 2023 | 110 | 1 | 0.009 | 0.00 | 0.05 |
| E Brito DV [14] | 2024 | 55 | 0 | 0.000 | 0.00 | 0.06 |
| Total | - | 522 | 6 | 0.011 | 0.00 | 0.02 |

CI, conﬁdence interval; LL, lower limit; UL, upper limit;

**Supplementary Table 7. Severe urinary incontinence complications.**

| First author | Year | Patients(n) | Urinary incontinence | Incidence (95% CI) | LL | UL |
| --- | --- | --- | --- | --- | --- | --- |
| Chin JL [48] | 2021 | 268 | 44 | 0.164 | 0.12 | 0.21 |
| Lian H [28] | 2016 | 32 | 1 | 0.031 | 0.00 | 0.16 |
| Vestris PG [20] | 2021 | 29 | 2 | 0.069 | 0.01 | 0.23 |
| Bomers JGR [49] | 2020 | 62 | 0 | 0.000 | 0.00 | 0.06 |
| Carbonell E [15] | 2024 | 77 | 3 | 0.039 | 0.01 | 0.11 |
| Tan WP [17] | 2023 | 110 | 0 | 0.000 | 0.00 | 0.03 |
| E Brito DV [14] | 2024 | 55 | 3 | 0.055 | 0.01 | 0.15 |
| Total | - | 633 | 53 | 0.084 | 0.06 | 0.11 |

CI, conﬁdence interval; LL, lower limit; UL, upper limit;

**Supplementary Table 8. Severe urinary tract infection complications.**

| First author | Year | Patients(n) | Urinary tract infection | Incidence (95% CI) | LL | UL |
| --- | --- | --- | --- | --- | --- | --- |
| Siddiqui KM [27] | 2016 | 157 | 0 | 0.000 | 0.00 | 0.02 |
| Lian H [28] | 2016 | 32 | 0 | 0.000 | 0.00 | 0.11 |
| Bomers JGR [49] | 2020 | 62 | 0 | 0.000 | 0.00 | 0.06 |
| Carbonell E [15] | 2024 | 77 | 0 | 0.000 | 0.00 | 0.05 |
| Tan WP [17] | 2023 | 110 | 0 | 0.000 | 0.00 | 0.03 |
| E Brito DV [14] | 2024 | 55 | 0 | 0.000 | 0.00 | 0.06 |
| Total | - | 493 | 0 | 0.000 | 0.00 | 0.01 |

CI, conﬁdence interval; LL, lower limit; UL, upper limit;

**Supplementary Table 9. Severe urinary retention complications.**

| First author | Year | Patients(n) | Urinary retention | Incidence (95% CI) | LL | UL |
| --- | --- | --- | --- | --- | --- | --- |
| Siddiqui KM [27] | 2016 | 157 | 0 | 0.000 | 0.00 | 0.02 |
| Lian H [28] | 2016 | 32 | 0 | 0.000 | 0.00 | 0.11 |
| Vestris PG [20] | 2021 | 29 | 0 | 0.000 | 0.00 | 0.12 |
| Bomers JGR [49] | 2020 | 62 | 0 | 0.000 | 0.00 | 0.06 |
| Carbonell E [15] | 2024 | 77 | 0 | 0.000 | 0.00 | 0.05 |
| Tan WP [17] | 2023 | 110 | 0 | 0.000 | 0.00 | 0.03 |
| E Brito DV [14] | 2024 | 55 | 0 | 0.000 | 0.00 | 0.06 |
| Total | - | 522 | 0 | 0.000 | 0.00 | 0.01 |

CI, conﬁdence interval; LL, lower limit; UL, upper limit;

**Supplementary Table 10. Severe urethral sloughing/stenosis complications.**

| First author | Year | Patients(n) | Urethral sloughing/stenosis | Incidence (95% CI) | LL | UL |
| --- | --- | --- | --- | --- | --- | --- |
| Siddiqui KM [27] | 2016 | 157 | 13 | 0.083 | 0.04 | 0.14 |
| Lian H [28] | 2016 | 32 | 1 | 0.031 | 0.00 | 0.16 |
| Bomers JGR [49] | 2020 | 62 | 1 | 0.016 | 0.00 | 0.09 |
| Carbonell E [15] | 2024 | 77 | 0 | 0.000 | 0.00 | 0.05 |
| Tan WP [17] | 2023 | 110 | 0 | 0.000 | 0.00 | 0.03 |
| E Brito DV [14] | 2024 | 55 | 0 | 0.000 | 0.00 | 0.06 |
| Total | - | 493 | 15 | 0.030 | 0.02 | 0.05 |

CI, conﬁdence interval; LL, lower limit; UL, upper limit;
